# Potential role of fecal microbiota from patients with slow transit constipation in the regulation of gastrointestinal motility

**DOI:** 10.1038/s41598-017-00612-y

**Published:** 2017-03-27

**Authors:** Xiaolong Ge, Wei Zhao, Chao Ding, Hongliang Tian, Lizhi Xu, Hongkan Wang, Ling Ni, Jun Jiang, Jianfeng Gong, Weiming Zhu, Minsheng Zhu, Ning Li

**Affiliations:** 1 Department of General Surgery, Jinling Hospital, Medical School of Nanjing University, Nanjing, 210002 China; 2 Center of Reproductive Medicine, Jinling Hospital, Medical School of Nanjing University, Nanjing, 210002 China; 3 Department of Medical Genetics, and Jiangsu Key Laboratory of Molecular Medicine, Medical School of Nanjing University, Nanjing, 210002 China; 4 First Affiliated Hospital, School of Medicine, Zhejiang University, Hangzhou, 310000 China; 5 Model Animal Research Center and MOE Key Laboratory of Model Animal for Disease Study, Nanjing University, Nanjing, 210002 China; 6 Shanghai Tenth People’s Hospital, Tenth People’s Hospital of Tongji University, Shanghai, 200072 China

## Abstract

The gut microbiota is involved in various physiological functions, and disturbances in the host-microbiome have been proven to contribute to the dysfunction of gut; however, whether microbiota participates in the pathogenesis of constipation remains unclear. In this study, we extracted and analyzed microbiota in feces from constipated donors who had undergone effective therapy with fecal microbiota transplantation, transplanted microbiota into pseudo-germ-free mice, and measured gut motility. These mice presented with lower pellet frequency and water percentage, smaller pellet size, delayed gastrointestinal transit time, and weaker spontaneous contractions of colonic smooth muscle. To determine the mechanism underlying delayed gut motility, microbial metabolites were measured. Short chain fatty acids and secondary bile acids were decreased in mice receiving microbiota from constipated donors. Moreover, the compositional changes of gut microbiota in constipated patients were identified, including the operational taxonomic unit, and the species richness and α diversity were much greater than those in healthy volunteers. These findings suggest that alterations of the microbiome might affect gut motility via altered microbial-derived metabolites in the development of constipation, and the restoration of disturbed microbiota might improve the clinical phenotype. This study indicates that regulating the intestinal environment may be a novel therapy strategy for constipation.

## Introduction

Chronic constipation is a functional bowel disorder with a high incidence worldwide, affecting 14% of adults and specifically almost 36% of the elderly^1^. According to Rome IV criteria, chronic constipation is characterized by various symptoms of difficult, infrequent, or incomplete defecation predominate^2^. There are three broad categories in chronic constipation including normal-transit constipation, slow-transit constipation (STC), and defecatory or rectal evacuation disorders^3^. Among these, STC is the majority category characterized by the markedly increased colonic transit time^3^.

Chronic constipation is a multifactorial disorder including a complex pathogenesis that still warrants further discussion. Earlier investigations have focused on the enteric nervous system (ENS) and interstitial cells of Cajal (ICC). In patients with STC, both the amount and morphology of ENS or ICC are altered, which may play a crucial role in the pathophysiology of colorectal motility disorders^4^. For instance, the myenteric plexus showed a reduced ganglionic density and size (moderate hypoganglionosis) compared with those in the control group^5^. In addition, colonic endocrine cells, which play a pivotal role in the regulation of colon motility, absorption, and secretion, were changed in STC^6^. There are also other studies reporting changes in autonomic dysfunction, morphologic changes in the myenteric and submucosal plexus, and reduced neurotransmitter levels (such as 5-HT, NO and VIP) in some patients with STC^2, 7^. However, these studies on STC can only explain some cases, and have focused less on the intestinal contraction itself. Therefore, it is also necessary to conduct research on the contractile property in constipation.

Recently, increasing studies have indicated that the gut microbiota plays a key role in health, and microbiota affects various activities of host physiology, including gut motility^8, 9^. The microbiota is dominated by bacteria belonging to the phyla Bacteroidetes, Firmicutes, and Actinobacteria. The microbiota inhabits the different regions of the digestive tract, and the colon is most densely populated^10^. Interestingly, accumulating evidence has demonstrated that the composition of the intestinal microbiota is different between constipated patients and healthy controls^11–14^. It was found that the composition of the colonic mucosal microbiota differed between constipated patients and controls, such that genera from *Bacteroidetes* were more abundant in the colonic mucosal microbiota of patients with constipation^15^. In the clinic, stool consistency is categorized by the Bristol Stool Scale (BSS) scores. Stool forms 1 and 2 are strongly associated with slower transit^2^. Vandaputte *et al*.^16^ reported that stool consistency was strongly associated with all known major microbiome markers. In their study, the microbiome markers were negatively related to species richness, while they were positively correlated with the Bacteroidetes:Firmicutes ratio, and negatively related to *Akkermansia* and *Methanobrevibacter* abundance. In addition, the abundance of methanogens (such as *Methanobrevibacter*) was increased in harder stools, a finding that was consistent with the elevated methane production in patients with chronic constipation, and methane was suggested to slow down gut motility^17^. These pathophysiological changes in constipated patients raise the reflections on whether striking changes in the fecal flora could be one cause of constipation. On the other hand, fecal microbiota transplantation (FMT) has been proposed as a therapeutic approach for functional gastrointestinal disorders by the reestablishment of the intestinal microenvironment. It was demonstrated that FMT was safe and effective for chronic constipation with clinical improvement and remission rates were 66.7% and 42.9% at our hospital^18^. However, these studies on the relationship between microbiota and gut motility did not discuss whether striking changes in the fecal flora are the cause of constipation. Therefore, it is necessary to perform systemic research on the role of microbiota in constipation.

It is well-documented about the role of gut microbiota in metabolism. Gut microbiota can impact stool habit, gastrointestinal transit, and stool weight^19^. There are two important luminal factors modulated by the microbiota, including short chain fatty acids (SCFAs) and bile acids (BAs). SCFAs were demonstrated to modify colonic motility through nerves and polypeptide YY release in rats^20^. Additionally, SCFAs could stimulate 5-HT_3_ receptors located on the vagal sensory fibers to release of 5-HT from enterochromaffin cells (ECs), thus accelerating colonic transit^21^. BAs, another microbial-derived metabolite, was revealed to stimulate the release of 5-HT and calcitonin gene-related peptide (CGRP) from ECs and intrinsic primary afferent neurons via activating TGR5, which led to the peristaltic reflex^22^. Interestingly, secondary BAs were proposed to directly act on ECs to synthetize 5-HT by elevating colonic TPH1 expression^23^. 5-HT was reported to induce the contraction of colonic myocytes due to the release of Ca^2+^ from the sarcoplasmic reticulum by activating 5-HT_3_ receptors and the inositol 1, 4, 5-trisphosphate pathway^24^.

To answer whether striking changes of gut microbiota between chronic constipation and health do harm to the host, we first selected six STC donors who received the clinical therapy of FMT effectively. Next, we transplanted fecal microbiota from donors to mice and monitored the symptoms of constipation. Because microbiota metabolism of dietary substrates has been speculated to be associated with gut motility^10, 25, 26^, we measured the related molecules, such as SCFAs and BAs, in mice to ascertain the possible mechanism of microbiota from STC in gut motility.

## Results

### Donors’ characteristics and improved efficacy of FMT in STC

**Figure 1:**
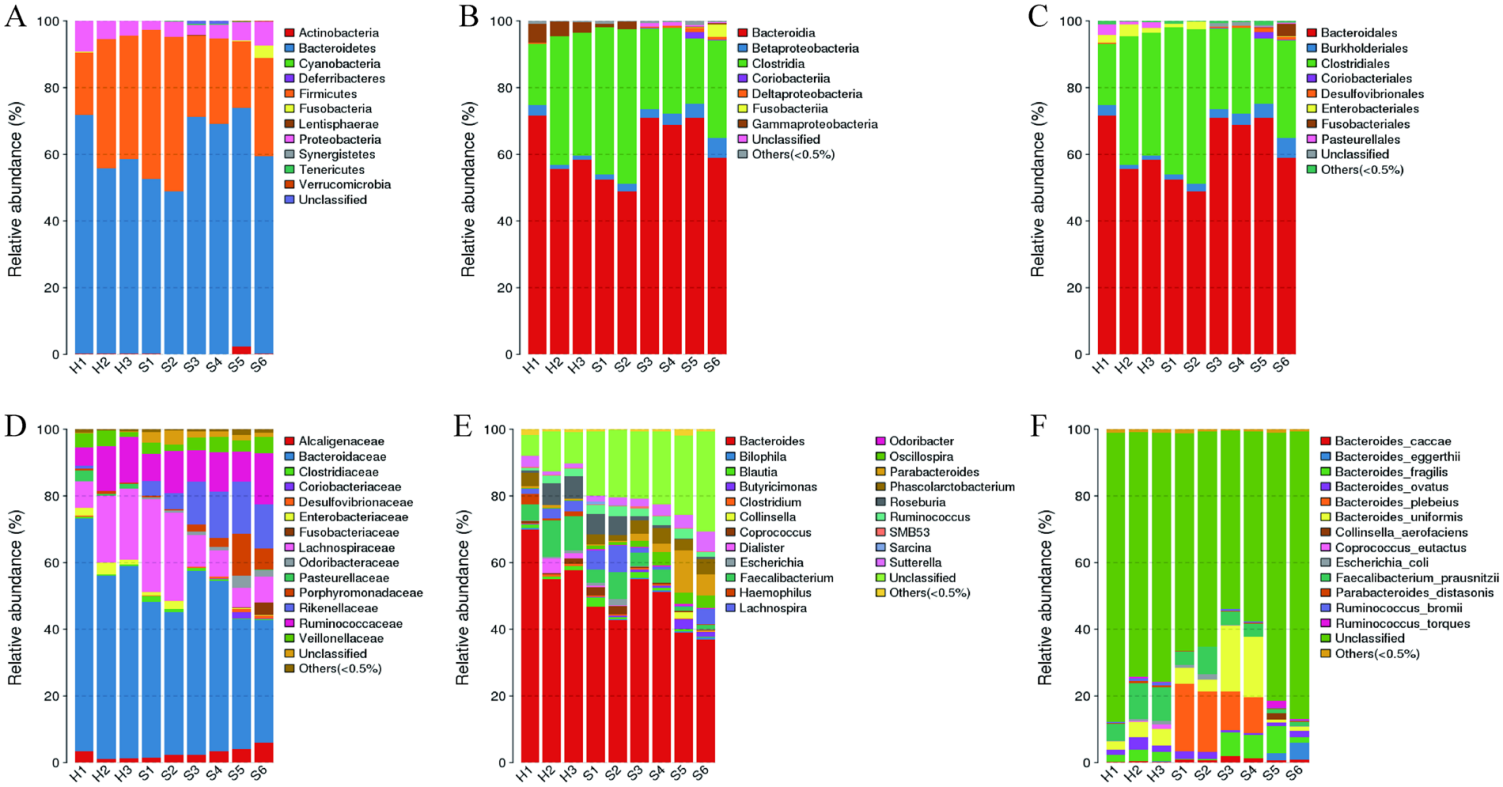
Microbiota composition of healthy donors and STC donors. (**A**) Phylum level; (**B**) Class level; (**C**) Order level; (**D**) Family level; (**E**) Genus level; (**F**) Species level. STC: slow transit constipation; Group H (n = 3): healthy donor; Group S (n = 6): slow transit constipation donor.

Six STC and three healthy donors were eligible for inclusion^18^. All of the enrolled STC donors received clinical therapy of FMT effectively. Their mean age was 57.2 years (range: 48–65 years), and the mean duration of constipation was 13.7 years (range: 6–20 years). The microbiota analyses showed that the operational taxonomic unit (OTU) of constipated patients was greater than that of healthy donors (180.2 ± 13.6 vs 123.7 ± 3.2, respectively; p = 0.021), with increased species richness (Chao index, 186.3 ± 14.2 vs 143.3 ± 10.9, respectively; p = 0.082) and α diversity (Shannon index, 3.54 ± 0.06 vs 3.06 ± 0.06, respectively; p = 0.006). The baseline demographics of the donors are presented in Table 1. The fecal microbiota of the healthy and STC donors presented a difference in their composition (Fig. 1). After treatment with FMT, it showed an increased stool frequency from 1.6 ± 0.2 to 5.0 ± 0.4 per week (p < 0.05), and an improved stool consistency from 2.0 ± 0.3 to 3.3 ± 0.2 (p < 0.05) in patients. In addition, the patient assessment of constipation symptoms questionnaire (PAC-SYM) and gastrointestinal quality-of-life index (GIQLI) scores were improved at 12 weeks after FMT. Compared with pre-treatment, colonic transit time (CTT) improved from 81.9 ± 9.5 to 53.3 ± 11.2 hours (p < 0.05). The details of the efficacy in STC patients receiving FMT are shown in Table 2.

**Table 1:** Clinical characteristics of included donors at baseline (Healthy donor = 3, STC donor = 6).

Donor No.	Sex (F/M)	Age (y)	Weight (kg)	Height (m)	BMI (18.5–25)	Disease Duration (y)	NO. BM Per Week Before Therapy
STC donor 1	F	55	60	1.65	22.0	15	1
STC donor 2	F	65	65	1.60	25.4	20	2
STC donor 3	M	64	68	1.68	24.1	18	2
STC donor 4	M	56	60	1.70	20.8	15	1.5
STC donor 5	M	55	55	1.60	21.5	8	1.5
STC donor 6	F	48	47	1.58	18.8	6	1.5
Health donor 1	F	22	50	1.60	19.5	0	7
Health donor 2	F	24	55	1.65	20.2	0	7
Health donor 3	F	23	60	1.62	22.9	0	7

F, female; M, male; BMI, body mass index; BM, bowel movement; STC, slow transit constipation.

**Table 2:** Outcome measures in constipated patients with fecal microbiota transplantation (n = 6).

Outcomes	Times	Results	P value
No. of BM/week	Pre-treatment	1.6 ± 0.2	<0.001
	12 week	5.0 ± 0.4	
Stool consistency score	Pre-treatment	2.0 ± 0.3	0.0025
	12 week	3.3 ± 0.2	
PAC-SYM score	Pre-treatment	2.0 ± 0.4	0.031
	12 week	1.5 ± 0.6	
GIQLI score	Pre-treatment	80.3 ± 11. 6	0.009
	12 week	122.5 ± 15.7	
CTT (hours)	Pre-treatment	81.9 ± 9.5	0.012
	12 week	53.3 ± 11.2	

BM, bowel movement; PAC-SYM, Patient Assessment of Constipation Symptoms; GIQLI, Gastrointestinal Quality-of-Life Index; CTT, colonic transit time.

### Composition of the intestinal microbiome in pseudo-germ-free mice

**Figure 2:**
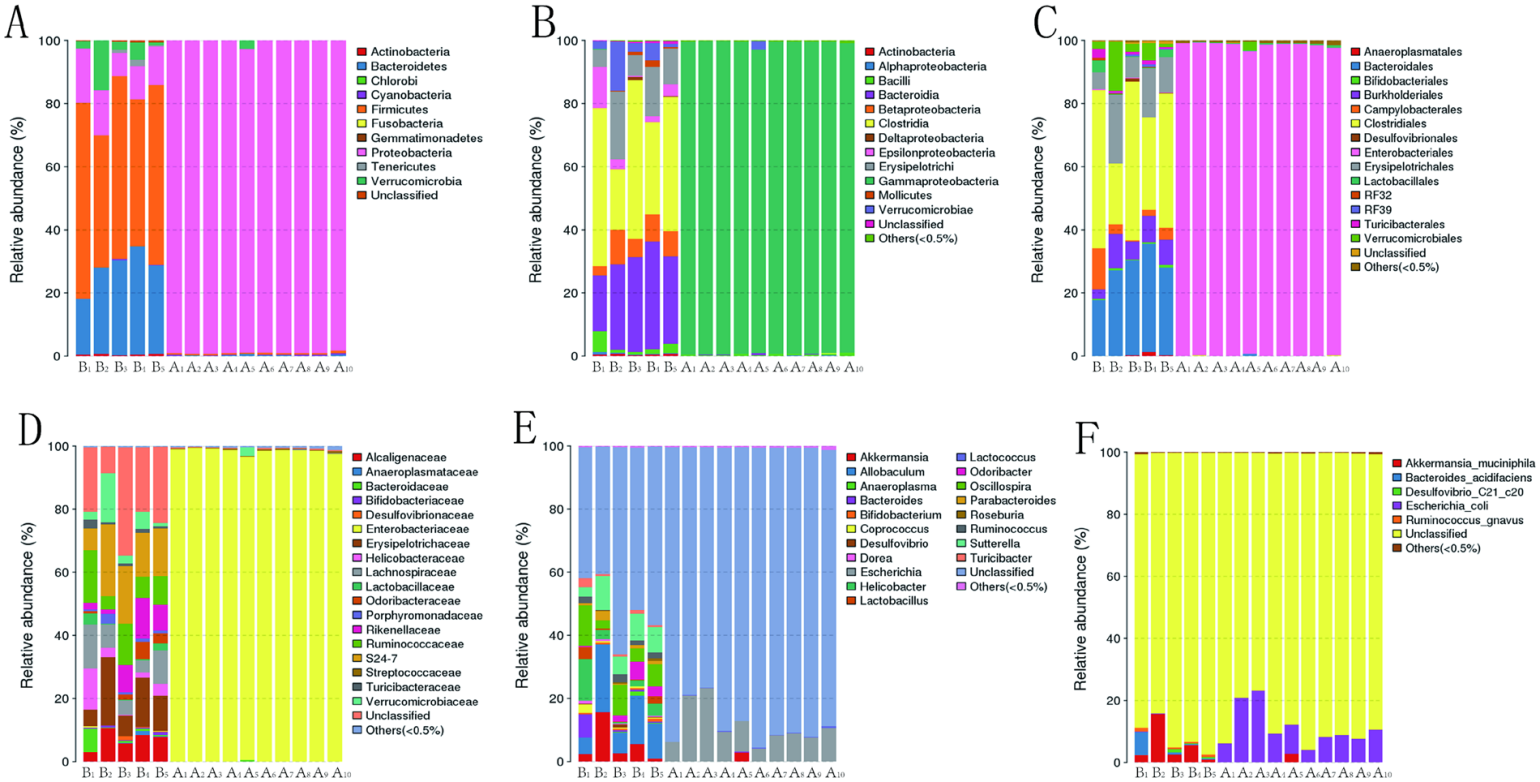
Microbiota composition of antibiotics-treated mice at every level. (**A**) Phylum level; (**B**) Class level; (**C**) Order level; (**D**) Family level; (**E**) Genus level; (**F**) Species level. Group B (n = 5): SPF mice without antibiotics; Group A (n = 10): SPF mice with antibiotics.

Pseudo-germ-free mice were produced by broad-spectrum antibiotics treatment for 4 weeks^27, 28^. After antibiotics combination, the composition of commensal bacteria was largely changed (Fig. 2). The OTU of mice decreased significantly from 341.0 ± 19.1 to 74.8 ± 2.6 (p < 0.001). Taxonomically, the abundance of most intestinal microbiota decreased significantly in all levels as shown in Fig. 2(A–F) (Phylum, Class, Order, Family, Genus, and Species). The species richness (Chao index) decreased from 354.3 ± 19.2 to 98.3 ± 4.8 (p < 0.001), and α diversity (Shannon index) decreased from 4.0 ± 0.2 to 0.5 ± 0.1 (p < 0.001). Because most of the resident intestinal microbiota was depleted, pseudo-germ-free mice could be used in mouse humanization studies when gnotobiotic facilities were limited.

### Fecal microbiota from the STC donors affects the fecal parameters, total gastrointestinal transit, and colonic transit in pseudo-germ-free mice

**Figure 3:**
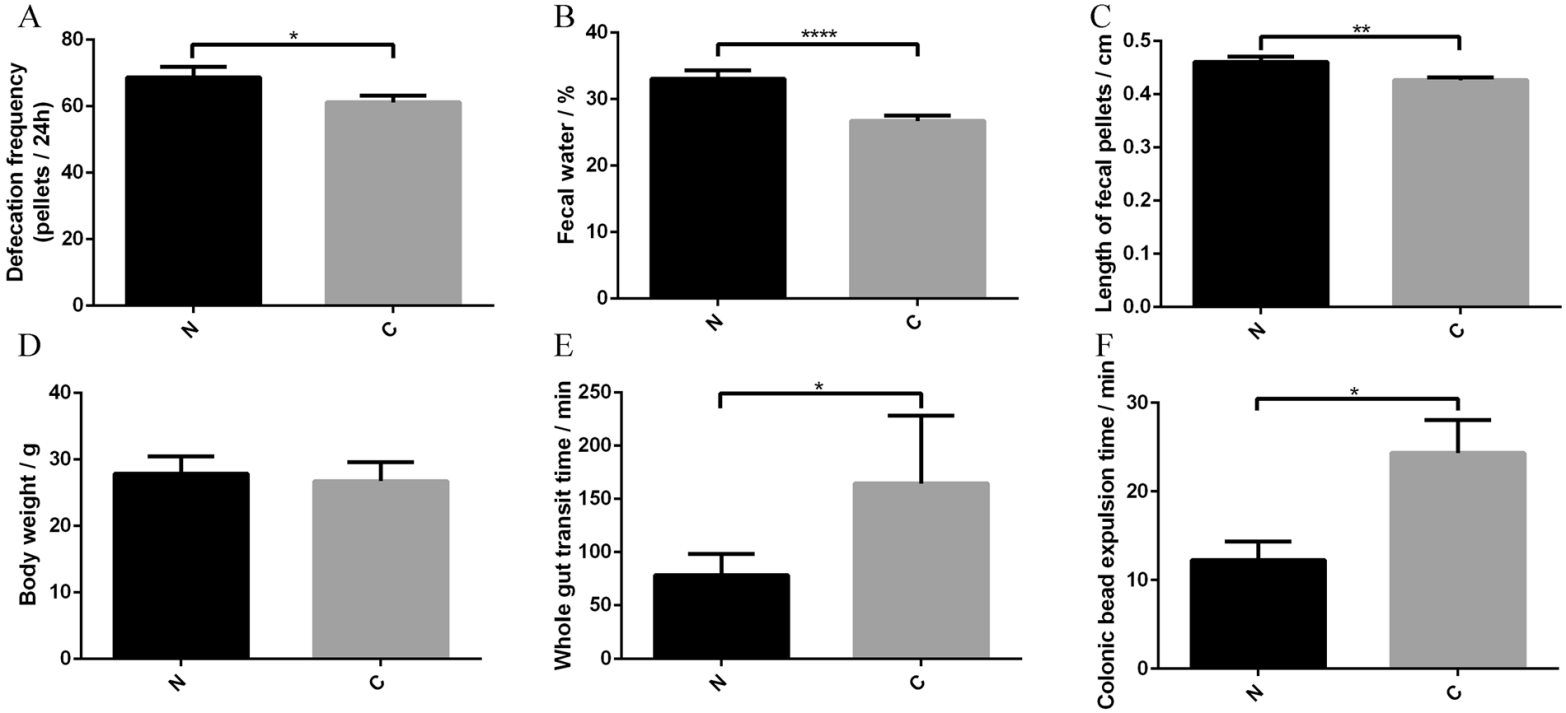
Transplantation of intestinal microbiota from STC donors has effects on defecation frequency (Group N = 20, Group S = 30) (**A**), fecal water content (Group N = 20, Group S = 30) (**B**), length of fecal pellets (Group N = 20, Group S = 30) (**C**), body weight after experiments (Group N = 20, Group S = 30) (**D**), total gastrointestinal transit time (Group N = 5, Group S = 10) (**E**), and colonic transit time (Group N = 5, Group S = 10) (**F**). Data represent the mean ± SEM. *p < 0.05, **p < 0.01, ***p < 0.001, ****p < 0.0001. Group N: mice receiving fecal microbiota from healthy donor; Group C: mice receiving fecal microbiota from STC donors. STC: slow transit constipation.

After 8 weeks by gavage with fecal microbiota from STC and healthy donors, both fecal parameters and gut motility were affected. Pseudo-germ-free mice receiving from STC donors had lower pellet frequency (68.6 ± 3.2 vs 61.1 ± 2.1 pellets/24 hours, p = 0.049), lower water percentage (33.0 ± 1.3 vs 26.7 ± 0.8%, p < 0.001), and smaller pellet size (0.46 ± 0.01 vs 0.42 ± 0.01 cm, p = 0.002) than mice receiving fecal microbiota from healthy donors (Fig. 3A–C). However, the body weight of the two groups was not different (27.8 ± 0.7 vs 26.7 ± 0.6 g, p = 0.214) (Fig. 3D). The total gastrointestinal transit time was greater in mice from STC donors, indicating significantly slower transit (78.0 ± 10.1 vs 164.2 ± 28.5 min, p = 0.037) (Fig. 3E). The colonic transit test was longer in mice from STC donors, indicating that fecal microbiota from STC donors caused slower colonic propulsion in mice (12.3 ± 2.1 vs 24.3 ± 3.7 min, p = 0.029) (Fig. 3F). Therefore, mice showed typical symptoms of constipation after receiving intestinal microbiota from STC donors.

### Contractility of proximal colonic muscle

**Figure 4:**
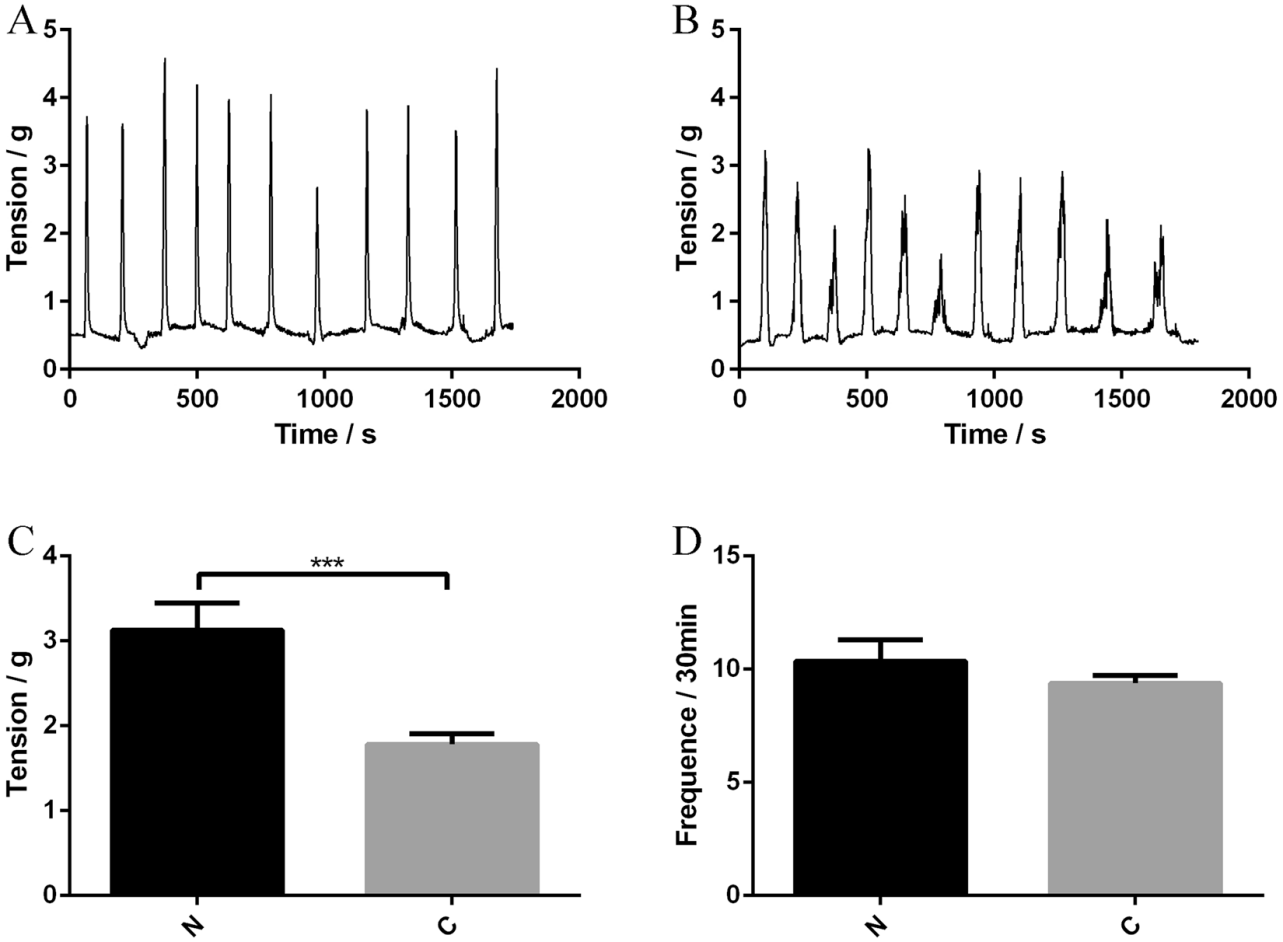
Transplantation of fecal microbiota from STC donors regulates colonic contractility (Group N = 6, Group S = 12). Recordings were made of spontaneous phasic contractions of proximal colon. Representative recordings from mice receiving fecal microbiota of healthy donors (**A**) and mice receiving fecal microbiota of STC donors (**B**). Tension (**C**) and frequency (**D**) normalized to basal values in mice receiving fecal microbiota from healthy and STC donors. Data represent the mean ± SEM. ***p < 0.001. Group N: mice receiving fecal microbiota from healthy donor; Group C: mice receiving fecal microbiota from STC donors. STC: slow transit constipation.

As pseudo-germ-free mice receiving fecal microbiota from STC donors had a decreased fecal output and delayed gut transit, it raised the possibility that the spontaneous contraction of intestinal smooth muscle might be affected by fecal microbiota from STC donors. After receiving fecal microbiota from STC donors, the average spontaneous phasic contractions of colonic longitudinal smooth muscle were inhibited. The tension decreased significantly from 3.12 ± 0.32 to 1.78 ± 0.12 g (p < 0.001), while the waveform did not change significantly (10.3 ± 1.0 vs 9.4 ± 0.3/30 min, p = 0.259) (Fig. 4). Therefore, microbiota from STC donors might inhibit the contractions of colonic smooth muscle in patients that could result in the lack of gastrointestinal motility.

### The host metabolism in the colon is influenced by fecal microbiota from STC donors

**Figure 5:**
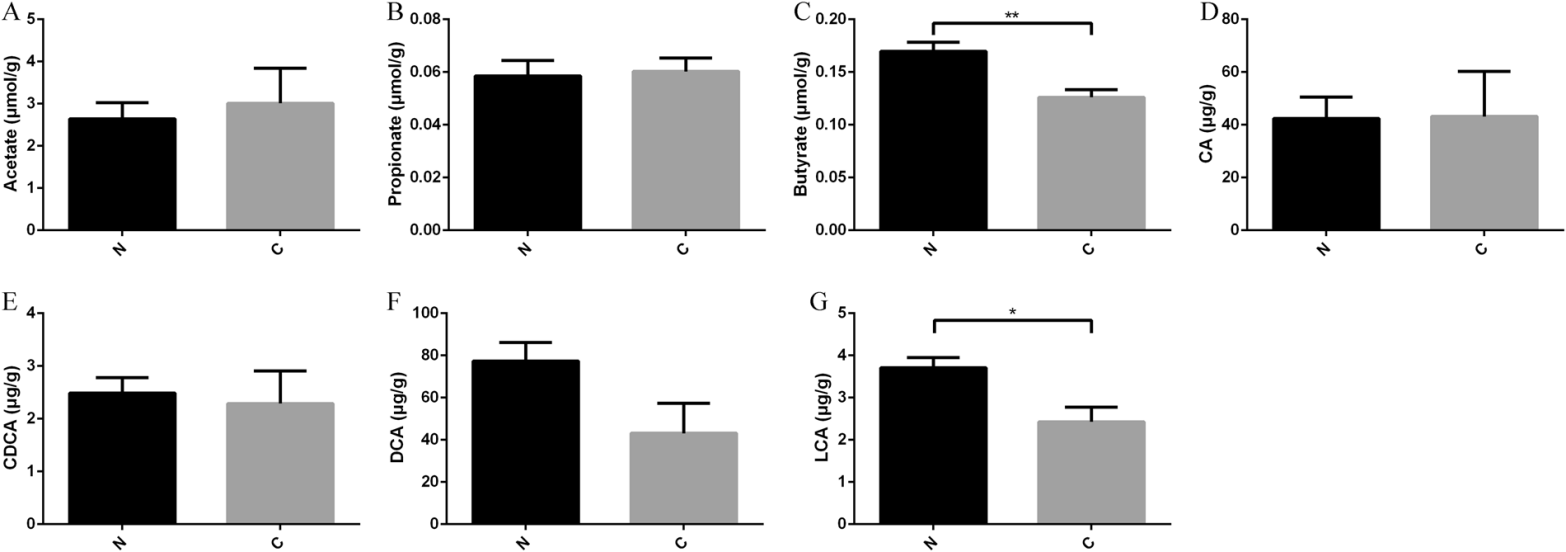
Transplantation of microbiota from STC donors altered host metabolism in colon (Group N = 5, Group S = 5). SCFAs (acetate, propionate and butyrate) (**A**–**C**), primary BAs (CA and CDCA), and secondary BAs (DCA and LCA) levels (**D**–**G**) change in mice receiving fecal microbiota from STC donors. Data represent the mean ± SEM. *p < 0.05, **p < 0.01. Group N: mice receiving fecal microbiota from healthy donor; Group C: mice receiving fecal microbiota from STC donors. STC: slow transit constipation; SCFA, short chain fatty acids; CA, cholic acid; CDCA, chenodeoxycholic; DCA, deoxycholic acid; LCA, lithocholic acid.

After 8 weeks of colonization with fecal microbiota from STC and healthy donors, SCFAs and BAs in the cecum were analyzed, respectively. SCFAs are generated in the colon as a result of the bacterial fermentation of dietary fiber and resistant starch, which mainly include acetate, propionate, and butyrate^29^. Bile acids are endogenous molecules synthesized from cholesterol in hepatocytes to form primary BAs, which are then metabolized by the gut microbiota to form secondary BAs^25, 30^. Our results showed that butyrate levels were decreased significantly in mice treated with STC donors (0.17 ± 0.01 vs 0.13 ± 0.01 μmol/g, p = 0.008) (Fig. 5). However, there was no significant difference in the levels of acetate (2.64 ± 0.4 vs 3.01 ± 0.8 μmol/g, p = 0.710) or propionate (0.058 ± 0.006 vs 0.060 ± 0.005 μmol/g, p = 0.840) (Fig. 5). The analysis of the BA levels showed that there was no significant difference in primary BAs (cholic acid (CA): 42.27 ± 8.23 vs 43.12 ± 17.12 μg/g, p = 0.966; chenodeoxycholic (CDCA): 2.48 ± 0.29 vs 2.28 ± 0.62 μg/g, p = 0.779) (Fig. 5), but there was a tendency towards decreased concentrations of secondary BAs. The deoxycholic acid (DCA) levels in mice from STC donors were lower than those in mice from healthy donors (77.23 ± 8.87 vs 43.04 ± 14.18 μg/g, p = 0.083), and the lithocholic acid (LCA) levels were also decreased significantly in mice from STC donors (3.70 ± 0.25 vs 2.42 ± 0.35 μg/g, p = 0.025) (Fig. 5). Therefore, microbiota from STC donors that affected gut motility might be due to changes in host metabolism, similar to SCFAs and BAs.

### Supplementation with butyrate and secondary BAs alter the motility in mice

**Figure 6:**
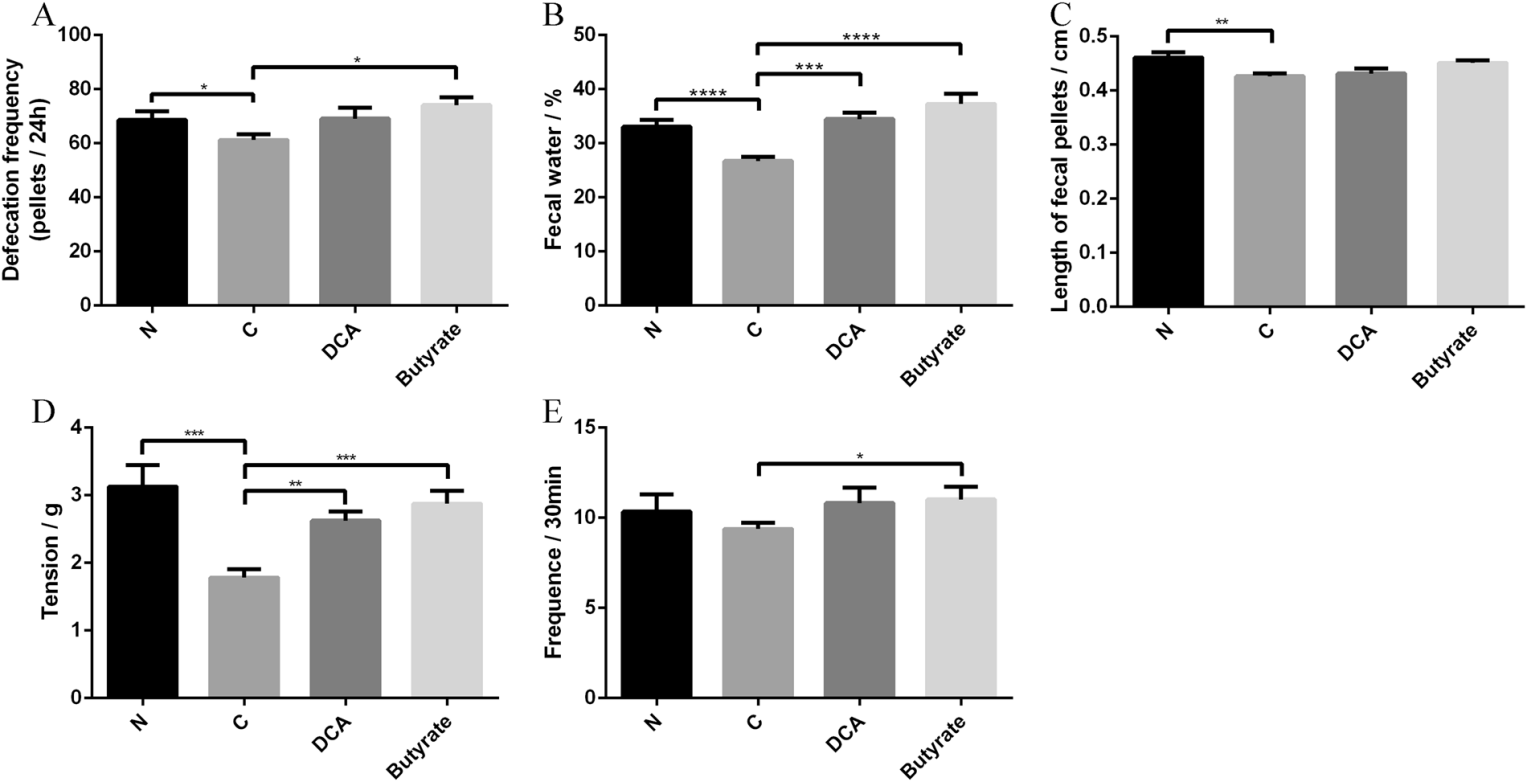
DCA and butyrate supplements have effects on defecation frequency (**A**), fecal water content (**B**), length of fecal pellets (**C**), and colonic contractility (**D**: tension; **E**: Frequency) of mice receiving fecal microbiota from STC donors (DCA mice = 6, Butyrate mice = 6). Data represent the mean ± SEM. *p < 0.05, **p < 0.01, ***p < 0.001, ****p < 0.0001. Group N: mice receiving fecal microbiota from healthy donor; Group C: mice receiving fecal microbiota from STC donors; Group DCA: mice receiving fecal microbiota from STC donors were treated with DCA; Group butyrate: mice receiving fecal microbiota from STC donors were treated with butyrate. STC: slow transit constipation; DCA: deoxycholic acid.

After supplementation with butyrate and DCA in drinking water for 2 weeks, the defecation frequency, fecal water content, length of fecal pellets and colonic contractility were examined in the mice (Fig. 6). Compared with mice from STC donors, DCA-treated mice had a higher water percentage (26.7 ± 0.8 vs 34.5 ± 1.2%, p < 0.001) and increased tension of contractility significantly (1.78 ± 0.12 vs 2.62 ± 0.13 g, p = 0.001), but there was no difference in the pellet frequency (61.1 ± 2.1 vs 69.0 ± 4.1 pellets/24 hours, p = 0.172), pellet size (0.42 ± 0.01 vs 0.43 ± 0.01 cm, p = 0.717) and frequency of contractility (9.4 ± 0.3 vs 10.8 ± 0.9/30 min, p = 0.078). In addition, butyrate-treated mice had a higher pellet frequency (61.1 ± 2.1 vs 74.0 ± 2.9 pellets/24 hours, p = 0.027), higher water percentage (26.7 ± 0.8 vs 37.2 ± 1.9%, p < 0.001), and increased tension (1.78 ± 0.12 vs 2.87 ± 0.19 g, p < 0.001) and frequency (9.4 ± 0.3 vs 11.0 ± 0.7/30 min, p = 0.031) of contractility, but there was no difference in the pellet size (0.42 ± 0.01 vs 0.45 ± 0.01 cm, p = 0.068).

## Discussion

It was gradually realized that establishing and maintaining a beneficial balance between intestinal microbiota and the human body are necessary for the normal functions of the intestine^9^. The dysbiosis will contribute to the pathogenesis of functional gastrointestinal disorders^10^. Therefore, it is essential to study the role of intestinal microbiota from STC patients in gut motility. In this study, the enrolled STC donors received FMT effectively, and the symptoms in STC patients were improved during follow-up. Next, pseudo-germ-free mice receiving fecal microbiota from STC donors were more likely to present constipation symptoms, including the hardness of stools, infrequent passage of stools, and poor gastrointestinal motility. In addition, microbiota metabolites in mice from STC donors were also changed in SCFAs and BAs. After supplementation with butyrate and DCA, some symptoms in mice from STC donors were reversed. This might be attributed to the disturbed intestinal microbiota that could change the host metabolism and affect gastrointestinal motility. Therefore, we concluded that aberrant gut microbiota composition in constipation might affect gut motility by altering host metabolism.

Commensal bacteria play a key role in the development and maintenance of intestinal sensory and motor functions^31^. Studies on germ-free mice showed that gastric emptying and gastrointestinal transit were significantly delayed compared with those in conventionally raised mice^31^. *Bacteroides thetaiotaomicron* was reported to stimulate gut motility by increasing the expression of γ-aminobutyric, vesicle-associated protein-33 and enteric γ-actin^32^. Meanwhile, *Lactobacillus acidophilus* and *Bifidobacterium bifidum* could accelerate intestinal transit by releasing neuromessengers, while *Micrococcus luteus* and *Escherichia coli* had an inhibitory effect^33^. After antibiotics, mice in the weekly fecal transfer treatments were fed by gavage for 8 weeks with fecal material from their respective donors. Using mice from STC donors, we herein showed that the symptoms of constipation could be replicated in mice. Recently, a growing body of work has implicated that the intestinal microbiota of constipated patients is altered^11–14^; for example, *unclassified_Ruminococcaceae*, *Alistipes*, and *Oscillibacter* were negatively correlated with the mean stool frequency^34^. Additionally, it has also been proven that human microbiota could promote the biosynthesis of 5-HT from colonic ECs to modulate gastrointestinal motility^23^. Therefore, our study indicated that normal microbiota might exert modulatory effects on gut motility.

A compelling set of relationships between microbiota metabolism and host physiology has emerged. SCFAs derived from the microbial fermentation of dietary fiber can directly inhibit histone deacetylases, activate G-coupled-receptors, and serve as energy substrates, thus affecting various aspects of physiological processes^35^. It has been reported that SCFAs stimulate the contractions of the intestine through an enteric cholinergic reflex^26^. Butyrate, one product of SCFAs, plays a strong regulatory role in microbial TLR-dependent sensing, which is implicated in gut motility by secreting PYY and GLP-1^36^. In our results, the butyrate levels were significantly lower in mice from STC donors than in mice from healthy donors. After supplementation with butyrate, the results of mice from STC donors were reversed in pellet frequency, water percentage, and colonic contractility. Therefore, fecal microbiota from STC donors might regulate gut motility by affecting the production of SCFAs.

Another particularly versatile class of microbial-produced metabolites, bile acids, is produced from cholesterol in hepatocytes and is metabolized by gut microbiota in the intestine^30, 37^. Evidence has demonstrated that BAs also impact gut motility by the release of serotonin from ECs^38–40^. When mice are transplanted with the microbiota from humans representing diverse culinary traditions, microbially deconjugated bile acid metabolites were correlated with faster gut transit^41^. Collectively, it is probable that BAs activate ECs to produce serotonin in the colon, and then alter gut motility. In our results, we found that the secondary BA level was lower in mice from STC donors, indicating that the microbial metabolism of BAs in the colon was disturbed. Decreased levels of secondary BAs could reduce the release of 5-HT from ECs, thereby affecting gut motility. After supplementation with DCA, the results of mice from STC donors were reversed in the water percentage and colonic contractility. However, the microbial metabolism of BAs is different between mice and humans. The primary BAs in humans are CA and CDCA, which in rodents are CA and muricholic acid (MCA). Next, deconjugated primary BAs that enter the colon are metabolized through 7-dehydroxylation into secondary BAs, including DCA from CA and LCA from CDCA. In rodents, the primary murine MCA also results in the formation of murideoxycholic acid (MDCA) in addition to DCA and LCA. In humans, ursodeoxycholic acid (UDCA) is a secondary BA that is formed by 7α/β-isomerization of CDCA, and this process can be performed by *Clostridium absonum* ^30, 42^. Therefore, altered bile acid profiles and a different metabolism process may affect various signaling through bile acid receptors between mice and humans. Thus, the results of our study warrant further discussion when translating findings from mice to humans.

FMT has shown efficacy for various gastrointestinal disorders, such as *Clostridium difficile* infection (CDI), inflammatory bowel disease (IBD), and irritable bowel syndrome (IBS). We previously suggested that FMT could increase bowel movements, improve the Wexner constipation score and GIQLI score in STC patients^18^. Studies have reported that in patients with recurrent CDI, the metabolism of bile salts and primary BAs to secondary BAs was disrupted, and FMT could result in the normalization of fecal bacterial community structure and could correct this abnormal metabolic composition^43^. FMT was also found to have a positive clinical response in ulcerative colitis, which was associated with successive colonization of donor-derived phylotypes^44^. Therefore, the mechanism of FMT might be the reestablishment of intestinal flora and abnormal microbiota metabolites in patients. Taken together, intestinal microbiota from STC donors was proven to affect gut motility, and the regulation of the intestinal microenvironment by FMT might be effective for constipated patients.

There are several limitations in this study. First, antibiotic treatment cannot completely remove the intestinal microbiota. Thus, it cannot be ruled out that some of the residual microbiota influence gut motility. Second, only six patients with slow transit constipation were enrolled, so we could not describe changes in the intestinal microbiota systematically. However, we plan to conduct a metagenomics analysis of intestinal microbiome in constipation in the future, with many more selected patients. Third, the observed changes in our study do not seem dramatic, possibly because gut motility is regulated by multiple signaling pathways. The intestinal microbiota might be just one of the causes. Thus, further work is necessary to clarify whether other pathways participate in the regulation of gut motility in constipation.

In summary, the results of this study have suggested that the colonization of the gut by microbiota from constipated donors might affect gut motility and stool consistency in mice through modulating host metabolism, and reestablishment of the intestinal microenvironment in constipated patients might be effective. The alterations of the microbiome in constipation, in turn, could affect the host, which might increase significant evidence for a novel treatment on fecal microbiota transplantation for constipated patients.

## Materials and Methods

### Healthy volunteers and STC donors

All procedures were conducted according to the National Institutes of Health Guide for the Care and Use of Laboratory Animals (NIH Publication No. 80-23), revised in 1996, and were approved by the Experimentation Ethics Review Committee of Jinling Hospital, Medical School of Nanjing University. Stool was collected from healthy donors that was previously used in a clinical fecal transplantation study in patients with STC, CDI, and IBD at our hospital^18, 45, 46^. Stool was collected and prepared from STC patients according to Rome IV (Table 1 for patients’ characteristics). The inclusion criteria and exclusion criteria were consistent with those in our previous study^18^. Fecal samples from both healthy donors and STC donors were collected for 16S ribosomal ribonucleic acid (rRNA) gene sequencing.

### Efficacy of fecal microbiota transplantation for STC donors

STC donors were treated according to the standardized FMT procedure at our hospital^18^. Spontaneous complete bowel movements (SCBMs) per week, stool consistency, PAC-SYM, GIQLI, and CTT were measured at the twelfth week after FMT^18^. The STC donors were enrolled in the following animal experiments only if there was clinical improvement or remission with FMT. Patients and their families signed the informed consent, and the study was approved by the human ethics committee of Jinling Hospital, Medical School of Nanjing University (2015NZKY-020).

### Animals

Male C57BL/6 mice (6 to 7 weeks old) provided by the Model Animal Research Center at Nanjing University were adapted to environmental conditions for one week before experiments. All animals were group-housed and fed the same autoclaved chow and water ad libitum under a 12-hour light/dark cycle, with a constant temperature of 21–22 °C and humidity of 55 ± 5%. The experiments followed the National Institutes of Health Guide for the Care and Use of Laboratory Animals (NIH Publication No. 80-23), revised in 1996, and were approved by the Experimentation Ethics Review Committee of Jinling Hospital, Medical School of Nanjing University.

#### Antibiotic treatment

Mice (n = 80) were provided with drinking water containing broad-spectrum antibiotics (ampicillin, 1 g/L; neomycin sulfate, 1 g/L; metronidazole, 1 g/L; vancomycin, 500 mg/L; Sigma-Aldrich, Shanghai, China) for 4 weeks ad libitum^47^. Antibiotics were renewed every two days. After 4 weeks, stool samples from the experimental mice were collected in a sterile micro-tube, and then were frozen immediately in liquid nitrogen and stored in a −80 °C freezer until analysis.

#### Microbiota Preparations for Mice Colonization

**Figure 7:**
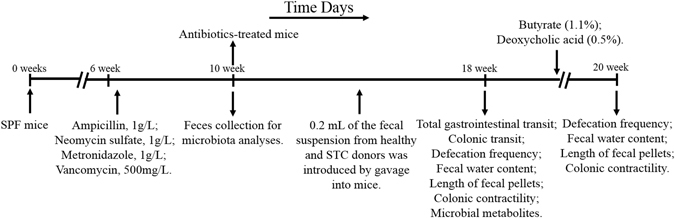
Experimental design indicating treatment and sampling times. STC, slow transit constipation.

Healthy and STC microbiota were prepared by diluting 1 g of fresh human fecal samples in 10 mL of reduced PBS under anaerobic conditions^48, 49^. The fecal material was then suspended by vortexing, and 0.2 mL of the suspension was introduced by gavage into each mouse recipient. After depletion of gut commensal microflora^27, 47, 50^, antibiotics-treated mice were given fecal microbiota from donors by gavage once a week for 8 weeks during the colonization experiments. After colonization, some mice from the same cohorts were then treated with SCFAs (1.1% sodium butyrate) or secondary BAs (0.5% sodium deoxycholate) in drinking water for two weeks (Sigma-Aldrich, Shanghai, China), with water renewed every two days^51, 52^. A diagram of the study design is shown in Fig. 7.

### Total gastrointestinal transit

After fasting overnight with free access to water, the mice were fed a semiliquid solution (0.1 mL) containing Evans blue (5%) (E2129, Sigma) and methyl cellulose (1.5%) (M0262, Sigma) by gavage^25^. After gavage, fecal pellets were monitored at 10 min intervals for the presence of the first blue pellet. The time for the expulsion of the first blue pellet was determined.

### Colonic transit

The mice were fasted overnight with free access to water. Colonic transit of mice was measured using a bead expulsion test^25, 53^. A 3-mm glass bead was inserted into the colon (2 cm proximal to the anal) using a plastic Pasteur lightly lubricated with lubricating jelly as described^25, 53^. The time until bead expulsion was measured.

### Fecal parameter and macroscopic measurements

Freely feeding mice were observed for 24 hours, and the number of pellets was counted every two hours. Fecal water content was measured by comparing the weight of the pellets at the end of the experiment and after drying (24 hours at 60 °C). Finally, fecal dimensions of each mouse were measured. In addition, animals were weighed every week from the beginning to the end of the experiments. The mice were sacrificed by isoflurane overdose, and the whole intestine (from stomach to anus) was excised.

### Analysis of smooth muscle contractility

Strips of longitudinal (6mm in length) muscle from the proximal colon were mounted in a 37 °C organ bath with a force transducer (MLT0202; AD Instruments, Spain) connected to a PowerLab (AD Instruments, Australia) recording device. Briefly, Ca2^+^-free HEPES-Tyrode (H-T) buffer (140.6 mmol/L NaCl, 2.7 mmol/L KCl, 1.0 mmol/L MgCl_2_, 10 mmol/L HEPES, and 5.6 mmol/L glucose, pH 7.4) was used to wash out the lumen contents of proximal colon segments. Next, the segments were transferred to the organ bath and equilibrated in H-T buffer (137.0 mmol/L NaCl, 2.7 mmol/L KCl, 1.0 mmol/L MgCl_2_, 1.8 mmol/L CaCl_2_, 10 mmol/L HEPES, and 5.6 mmol/L glucose, pH7.4) for 15 minutes. The resting tension was set to approximately 0.5 g prior to force measurement. The isotonic contractions of longitudinal muscle were recorded for 1 hour, with H-T buffer being changed every 15 minutes. The mean amplitude of the basal tension and frequency of phasic contractions were measured for 30 minutes after experiments. The detailed force assays were performed according to our previously described methods^54, 55^.

### Short chain fatty acid and bile acid determination in the cecum

The caecal contents from mice were collected, freeze-dried, and pulverized. Feces samples (150 mg) were homogenized and centrifuged in 1000 μL of 0.005 M aqueous NaOH that contained an internal standard (5 μg/mL caproic acid-d3). Next, SCFAs (acetate, propionate and butyrate) were quantified as in our previous study by gas chromatography-mass spectrometry (GC-MS) analysis^56^. The Agilent 7890A gas chromatography system coupled to an Agilent 5975 C inert XL EI/CI mass spectrometric detector (MSD; Agilent Technologies, Santa Clara, CA) was used to analyze the SCFA concentrations. BAs were quantified using liquid chromatography-tandem mass spectrometry (LC-MS/MS) as described previously^57^. Fecal samples (200 mg) were used for the BA determination (CA, CDCA, DCA and LCA). BA stock solutions were diluted with 50% methanol and spiked with internal standards to construct standard curves. The LC-MS/MS system consisted of an API 4000 QTrap (AB Sciex, Darmstadt, Germany) coupled to electrospray ionization. Chromatographic separation was achieved using an Agilent 1200 HPLC system (Agilent, Waldbronn, Germany). Each SCFA and BA was identified by their relative retention time compared with that of standard SCFAs and BAs, respectively.

### Microbiota analyses

Stool samples (500 mg) from mice or humans were collected in sterile tubes and were frozen immediately in liquid nitrogen and stored in a −80 °C freezer until processing for DNA isolation. The microbiota were analyzed using 16S rRNA pyrosequencing technology at the BGI laboratory (Beijing Genomic Institute, Shenzhen, China) as previously described^56^. Total DNA was isolated using a Wizard Genomic DNA Purification Kit according to the manufacturer’s instructions (Promega, Madison, USA). After DNA isolation, concentration testing and sample integrity were performed for sample analyses. A fluorometer or microplate reader was used to detect the concentration, and 1% agarose gel electrophoresis (voltage, 150 V; electrophoresis time, 40 minutes) was used to detect the sample integrity. Primers were designed to amplify the V4 hypervariable region according to the manufacturer’s recommendations^58^. Genomic DNA was normalized to 30 ng per polymerase chain reaction (PCR) for the library using the V4 Dual-index Fusion PCR Primer Cocktail and PCR Master Mix (NEB Phusion High-Fidelity PCR Master Mix; NEB, Ipswich, MA). The library validation was quantified through real-time quantitative PCR (qPCR) (EvaGreen; Biotium, Fremont, CA). The qualified libraries were sequenced pair end on the Illumina MiSeq platform (Illumina, San Diego, CA) using the sequencing strategy PE250 (MiSeq Reagent Kit; Illumina). Bioinformatics analysis was performed as previously described^56, 59^. The MOTHUR v.1.27.0 Standard Operation Procedure (SOP) and QIIME pipeline were used to analyze high-quality sequence reads.

### Statistical analysis

GraphPad Prism V.6.0 (San Diego, CA, USA) was used for all analyses and preparation of graphs. Continuous data were presented as the means ± SEM or median (range), while the categorical data were presented as n (%). Student’s *t*-test or the Mann-Whitney U-test was used to analyze continuous variables depending on the normality of the data distribution. Pearson’s chi-squared test or Fisher’s exact test was used for categorical variables as appropriate. Significant differences identified using the above tests were indicated in figures as follows: *p < 0.05, **p < 0.01, ***p < 0.001, ****p < 0.0001.
